# Understanding public perception of health examination services: Key factors influencing satisfaction in Türkiye

**DOI:** 10.1371/journal.pone.0324125

**Published:** 2025-05-29

**Authors:** Kübranur Çebi Karaaslan, Hülya Diğer, Tubanur Çebi

**Affiliations:** 1 Department of Econometrics, Faculty of Economics and Administrative Sciences, Erzurum Technical University, Türkiye; 2 Department of Health Management, Faculty of Economics and Administrative Sciences, Economic and Social Research Application and Research Center Manager, Erzurum Technical University, Türkiye; 3 Department of Dental Prosthesis Technology, The Health Services Vocational School, Atatürk University, Erzurum, Türkiye; Necmettin Erbakan Üniversitesi: Necmettin Erbakan Universitesi, TÜRKIYE

## Abstract

The aim of this study is to evaluate the level of satisfaction with health examination services in Türkiye. It is thought that the findings will contribute to the more effective management of the health service process and offer potential solutions to identified problems. Notably, a significant portion of the problems encountered in healthcare services tends to arise during the examination phase. Therefore, this research was conducted to address these problems by thoroughly analyzing public satisfaction, with the expectation that such an approach could provide actionable insights for resolving these problems. In the study, the micro data set of the 2023 Life Satisfaction Survey conducted by the Turkish Statistical Institute was used. The analysis process was carried out with a two-stage method. In the first stage, Pearson’s χ² test was used to evaluate whether the independent variables had a statistically significant relationship with satisfaction with health examination services. In the second stage, a considering the binary categorical structure of the dependent variable, a logit regression model was applied to estimate the relationship between satisfaction with health examination services and the independent variables. The findings revealed that 61.85% of Turkish citizens were satisfied with health examination services. Furthermore, this level of satisfaction was significantly affected by a wide range of sociodemographic, individual, and institution-related factors. The study’s findings suggest that aligning individuals’ demands in the health service process with guidance from field experts and developing targeted policies could lead to improved satisfaction with health examination services. In addition, it is foreseen that the concept of trust is important in the satisfaction that constitutes the main subject of the study in health services and in the negative situations experienced in different subjects. Based on these insights, initiatives can be taken to increase trust in the health system through health policies to be designed. Furthermore, the results highlight the growing importance of digitalization and digital hospitals in healthcare. Further progress in this direction will increase the satisfaction with health examination and contribute to positive results in health services.

## Introduction

Health services are a specialized type of service designed and delivered based on certain factors, especially the needs of the addressed segment. In this context, health demand emerges in line with needs and preferences. Healthcare institutions, as the providers of these services, perform many functions that are thought to significantly affect individuals’ preferences. Medical, patient care and hospitality functions of health institutions are among these functions.

The medical functions of healthcare institutions include services such as diagnosis, treatment, rehabilitative care, and procedures performed in operating rooms, polyclinics, and clinics, while patient care functions aim to meet patients’ specific needs [1]. Similarly, the hospitality function focuses on ensuring patient comfort, accommodation, and overall well-being throughout their healthcare experience [1]. In addition to these functions and concepts such as quality, the waiting time for the examination and the health personnel providing the examination also play a decisive role in the satisfaction of health services. In this context, studies on appointment density in Türkiye’s health system (The letter sent to private hospitals by the Social Security Institution in 2022 [2]; the transition to the approved appointment period in 2024 [3] play an important role in shaping examination services and thus increasing satisfaction. When the differences in health systems regarding the increase in satisfaction in health services are evaluated globally, it is predicted that the obligations for primary health care services (Germany, Slovenia, England, etc.) may contribute to the increase in satisfaction level by enabling the examination process to function correctly. In this context, many studies in the literature support this prediction.

In Taiwan, individuals aged 40–59 and those with university-level or higher education report greater satisfaction with hospital services [4]. Studies conducted in Malawi and Morocco indicate that women tend to express higher satisfaction with healthcare services [5,6]. In Canada, the perception of social isolation has a significant impact on satisfaction levels [7]. In Saudi Arabia, there is a statistically significant relationship between satisfaction and examination [8]. In Australia, telehealth services are a key contributor to satisfaction with healthcare services [9]. In Türkiye, mothers demonstrate high levels of satisfaction with health services [10]. In Sri Lanka, key performance indicators play a crucial role in ensuring the effective delivery of healthcare services [11]. In California, limited health literacy is associated with lower treatment satisfaction [12]. In Thailand, satisfaction was found to be high for serious and uncommon diseases [13]. In Indonesia, the waiting time for an examination and the duration of the examination itself play a significant role in determining satisfaction levels [14]. In Türkiye, integrating language and culture into health services has been found to affect satisfaction levels [15]. In Tanzania, key factors contributing to satisfaction with health services include how individuals feel, the quality-of-service delivery, and the ability to ask questions [16]. It is thought that the differences in the results obtained in the studies may be in a direct relationship with the health systems of the countries. In addition to health systems, a review of the literature reveals that many factors affect satisfaction with health services. Studies on healthcare satisfaction highlight that, beyond socio-demographic and socio-economic characteristics, factors such as examinations [8,14], service delivery [8,16], counseling [8], telehealth [9], waiting time for examinations [10,14], anxiety [10], health literacy [12], pharmacy services [13], alternative health insurance [14], quality [15], patient communication [15], and cultural factors [15] are frequently associated with satisfaction in health services.

It is also supported by the literature that many factors play an important role in satisfaction in health services. Although the relevant studies are conducted in different countries, it is seen that the health system plays a common role in the level of satisfaction. In this context, globally, there are many obstacles to access to health services due to various reasons, especially financing in the historical process. As a matter of fact, the World Health Organization’s adoption of the goal of “Health for All” in 2000 and its invitation to its members on this issue are among the initiatives to remove financial barriers to access to health. Similarly, in 1998, the World Health Organization stated in the “World Health Declaration” that certain principles are important in the health service delivery process. Necessary integration and feedback between health services are among the relevant principles [17]. In this regard, health policies in Turkey are designed by taking into account various factors (socio-demographic factors, socioeconomic factors, transportation, location, etc.) regarding access to health services and aim to increase satisfaction. As a matter of fact, the fact that the location of health institutions is determined in line with various regulations [18–20] supports this goal. Despite these improvements in health services, various deficiencies and failures may occur in the service process. In this regard, various reforms are being made and existing problems are tried to be solved.

On the other hand, challenges such as appointment density, prolonged waiting times, and rising incidents of violence in healthcare settings in Türkiye have prompted various changes and reforms in the health system. These reforms aim to address and resolve issues in healthcare delivery through targeted initiatives and policies. However, the presence of dissatisfied patients and their perceptions of healthcare may lead to problems that negatively affect not only their own health outcomes but also those of other patients. Therefore, this study was conducted to explore the concept of satisfaction with health examinations, with the goal of addressing these issues and turning negative outcomes into positive ones. The mentioned issues point to the original aspect of the study. In addition, the fact that the study focuses on the post-pandemic period and the factors that create an impact as well as the differentiated health perception and service demand in this sense are among the other unique aspects of the research. Based on the findings and results, the study aims to evaluate satisfaction with health examination services and provide recommendations for improving the health system in this regard. In this context, the study specifically examined satisfaction with health examination services.

## Literature review

The concept of quality in health constitutes the basis of thoughts on health services. In this sense, quality thinkers (Crosby, Feigenbaum, Ishikawa, Deming etc.) and various quality models (SERVQUAL, Andersen’s behavioral model etc.) play an important role in ensuring quality in health services.

The SERVQUAL model developed by Parasuraman, Zeithaml and Berry is used to determine the difference between people’s expectations and perceptions of quality [21]. Andersen’s behavioral model examines the factors in access to health services under certain headings [22]. In line with both national and international factors, various initiatives are being taken to provide quality services in Turkey’s health institutions. In particular, disadvantaged individuals (disabled, elderly, children, etc.) are taken into consideration to facilitate the service process and improve quality. In this context, various versions are being implemented and improvements are being made in Turkey’s hospitals in line with health quality standards. In the current process, Turkey’s hospitals use version 6.1 of health quality standards. There are various studies in the literature on the related subject.

The literature on medical examination services in healthcare encompasses studies addressing a range of variables and topics, employing diverse methodological approaches. When the literature is examined, it is evident that researchers have utilized various methods to explore the subject. These include the scheffe test [4], regression analysis [5,7–9,12–15,23], the fisher exact probability test [24], the one-sample t-test [11], correlation analysis [10], and content analysis [25,16]. Table 1 provides a general framework for studies conducted on satisfaction with health examination services.

**Table 1:** Summary of studies on satisfaction with health examination services.

Study	Country (Location)	Method	Data	Result
Ho (2009)	Taiwan	Scheffe test	929 survey data	According to the research results, people between the ages of 40–59 and those with a university or higher education level have higher hospital satisfaction.
Maseko et al. (2014)	Malawi	Logistic regression	Female patients examined between July and October 2013	As a result of the study, it was determined that women were satisfied with the services offered for cancer screening.
Mohammed et al. (2014)	Nigeria	Fisher Exact Probability Test	Random interviews with 147 healthcare institution staff	According to the research results, it was determined that health care organizations were generally satisfied with the optimal resource utilization activities in the health insurance program.
Selmouni et al. (2015)	Morocco	Cross-sectional study	Face-to-face interview with 324 women who participated in visual examination with acetic acid	According to the results of the study, most women think that visual examination with acetic acid can save lives. Other results obtained include that the majority of women participating in the study were satisfied with the service they received from health institutions.
Krupinski et al. (2017)	–	Evaluation of chest radiographs in two ways (with/without nodular addition) by 20 faculty members, 10 teaching assistants and 10 students	64 CR chest cases	According to the study results, fatigue does not change the effect of satisfaction, but it can contribute to it. In addition, satisfaction reduces both true-positive and false-positive results. On the other hand, fatigue was determined to reduce true-positives more than false-positives.
Hitzig et al. (2021)	Canada	Regression analysis	Survey data on 5,231 patients	According to the research results, it was determined that the perception of social isolation has an effect on satisfaction.
Alhaqbani and Bawazir (2022)	Saudi Arabia	Regression analysis	646 pregnant women in 11 health centers	. Other results obtained include:
Charters et al. (2022)	Australia	Regression analysis	Survey data of 124 patients	According to the research results, it has been determined that the provision of auxiliary health services via telehealth plays a role in achieving high satisfaction rates in health services.
Efe et al. (2022)	Türkiye	Correlation analysis	Mother of 316 children receiving inpatient treatment in the emergency room	According to the study results, mothers have a high level of health satisfaction. In addition, education level, anxiety and waiting time for examination are among the other results obtained that affect mothers’ health satisfaction.
Ginthotavidana and Waidyasekara (2022)	Sri Lanka	One sample t test	46 key performance indicators to determine the performance of housekeeping departments	Research results show that key performance indicators have an important place in the effective implementation of health services.
Mefford et al. (2022)	California	Regression analysis	Survey data of 2,154 patients	According to research results, limited health literacy is associated with lower treatment satisfaction.
Parinyarux and Yotsombut (2022)	Thailand	Regression analysis	Survey data collected via online social media between June and August 2021	It has been determined that patients’ general satisfaction with pharmacy services is high when it comes to serious and uncommon diseases.
Sakti et al. (2022)	Indonesia	Regression analysis	Survey data from 269 patients	According to the research results, the duration of the examination and waiting times have a determining role on satisfaction. In addition, among the other results obtained, the provision of alternative financing health insurance for patients who do not have health financing also plays a role on satisfaction.
Zikusooka et al. (2022)	Türkiye	Regression analysis	Survey data from 4,548 patients	According to the study results, the integration of health services with language and culture in refugee environments affects patient satisfaction in patient communication and service quality.
Heri et al. (2023)	Tanzania	Regression analysis	338 pregnant women	According to the results of the research, women are satisfied with the care of the service providers. On the other hand, while the ability to ask questions, how one feels and the provision of health services play the most role in satisfaction, the least determining role is played by parking, timing, waiting times, examinations and facilities and tests among the other results obtained.
Kodjebacheva et al. (2023)	–	Content analysis	14 studies on pediatric patient satisfaction	According to the research results, satisfaction with telehealth services was higher than face-to-face services in 9 out of 14 studies. However, telehealth services received more positive ratings than face-to-face visits.

Berbaum et al. (2000) examined the impact of erroneous decisions in chest radiography on satisfaction [26]. Ho (2009) investigated satisfaction with health institutions [4], while Maseko et al. (2014) focused on patient satisfaction with cervical cancer screening [5]. Mohammed et al. (2014) investigated satisfaction with the level and type of resources in the utilization of health insurance programs [24]. Selmouni et al. (2015) investigated satisfaction among cancer patients [6]. Krupinski et al. (2017) investigated the impact of fatigue on search satisfaction in the field of chest radiography [27]. Myszewski and Sinha (2020) analyzed the significance of patient satisfaction in healthcare [28], while Hitzig et al. (2021) investigated health and life satisfaction in adults [7]. Alhaqbani and Bawazir (2022) investigated satisfaction with antenatal services among pregnant women [8], and Charters et al. (2022) investigated patient satisfaction with telehealth services [9]. Efe et al. (2022) focused on mothers’ satisfaction with healthcare services [10], whereas Ginthotavidana and Waidyasekara (2022) examined satisfaction with health facilities [11]. Magadi and Magadi (2022) addressed ethnic disparities in patient satisfaction within primary healthcare [23], while Mefford et al. (2022) linked health literacy to treatment satisfaction [12]. Parinyarux and Yotsombut (2022) evaluated satisfaction with pharmacies [13], and Sakti et al. (2022) investigated patient satisfaction [14]. Zikusooka et al. (2022) investigated patient satisfaction in refugee health centers [15]. Heri et al. (2023) investigated women’s satisfaction with antenatal services [16]. Kim and Cho (2023) investigated the health examination service process in older adult patients [29]. Kodjebacheva et al. (2023) investigated satisfaction with telehealth services [25]. Studies in the literature on satisfaction with health examination services have been conducted in many countries. In this regard, studies on health examination services have been conducted in Taiwan [4], Malawi [5], Nigeria [24], Morocco [6], Canada [7], Australia [9], Türkiye [10], Sri Lanka [11], California [12], Indonesia [14], Thailand [13], Türkiye [15], Tanzania [16] and South Korea [29].

Studies in the literature on satisfaction with health examination services have been conducted in many countries. In this context, research on health examination services has been carried out in Taiwan [4], Malawi [5], Nigeria [24], Morocco [6], Canada [7], Australia [9], Türkiye [10], Sri Lanka [11], California [12], Indonesia [14], Thailand [13], Türkiye [15], Tanzania [16] and South Korea [29]. Considering the literature, it is evident that evaluating satisfaction with health examination services and the factors affecting satisfaction is of significant importance.

## Methods

### Data source

This study utilizes the micro data set from the 2023 Life Satisfaction Survey (LSS). The Data Sources section should primarily focus on the dataset and methodology rather than its historical background.

The Life Satisfaction Survey microdata used in this study was obtained from the Turkish Statistical Institute (Turkstat) through the official data request process. Researchers can apply for access to similar data through the Turkstat Research Data Portal, provided that approval is obtained in accordance with the Turkish Statistical Law No. 5429. These data are obtained from Turkstat in return for a contract without the need for an ethics committee document and are used in studies without additional permission [30]. In addition, the Turkish Statistical Institute has received a “Letter of Commitment” from the authors authorizing the use of the data for the study.

The main purpose of the LSS is to measure the individual’s general perception of happiness, social values, general satisfaction in basic life areas (health and social security, education, work life, income, personal security and justice services, hope for the future) and satisfaction with public services and to monitor changes in these satisfaction levels over time. Household members aged 18 and over living within the borders of the Republic of Turkey were included in the survey study. The sampling method of the study is two-stage stratified cluster sampling. The sample size of the study was designed to provide an estimate at the level of the sum of the Turkish Population Weights. Weighting was performed on the dataset obtained as a result of the sampling since selection probabilities were used due to the multi-stage sampling design. For the 2023 survey, the number of interviewees was 9,595, and the number of households surveyed was 4,659 [30].

### Measures and variables

**Fig 1:**
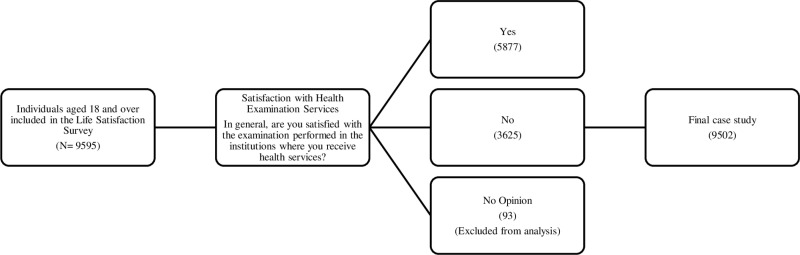
Sample selection scheme.

The dependent variable of the study is individuals’ satisfaction with health examination services. This variable was measured using the question: *“In general, are you satisfied with the examination performed in the organizations where you receive health services (yes; no; no opinion)?”* Within the study, responses indicating satisfaction were coded as 1, while those indicating dissatisfaction were coded as 0. Observations with “no opinion” were excluded from the analysis. Fig 1 illustrates the sample selection process.

After the literature review, chi-square independence tests were conducted to include variables that may affect the dependent variable in the data set. Finally, an independent variable set was created under the headings of sociodemographic, individual and institutional indicators. The definitions of the variables in the study are presented in Table 2.

**Table 2:** Variable definitions.

Variables	Description
*Dependent Variable*	
Satisfaction with Health Examination Services	Are you generally satisfied with the examination performed in the institutions where you receive health services?
*Independent Variables*	
Gender	Gender of the individual
Age	The age at which the individual graduated
Marital Status	Marital status of the individual
Education Level	The last diploma the individual received
Working Status	Individual’s working status
SSI Registration	Are you registered with any social security institution?
Income Satisfaction	Are you satisfied with your monthly household income?
Happiness Level	How happy are you when you consider your life as a whole?
Future Expectations	How do you think your life will be in general in the coming year?
Health Status	In the last year, I have had a serious health problem.
Interest in Health Issues	How interested are you in health-related issues?
Electronic Public Services Use Case	Do you benefit from public services provided electronically?
General Healthcare Service Satisfaction	Are you satisfied with the health services?
SSI Services Satisfaction	Are you satisfied with the services of the Social Security Institution?
Appointment Problem	Do you generally have problems making appointments for examinations and/or tests at the institutions where you receive health services?
Hygiene Problem	In general, are there any problems with cleanliness/hygiene in the institutions where you receive health services?
Problem Related to Doctor	In general, are there any problems with the way doctors treat patients in the institutions where you receive health services?
Adequacy of Number of Health Personnel	In general, do you find the number of doctors and healthcare personnel in the institutions where you receive healthcare services sufficient?
High Test and Examination Fees	Do you generally find the examination and test fees of the institutions from which you receive health services high?
The Problem of Drug Prices	Do you see any problems with drug prices in the institutions where you receive health services?
Queue Waiting Problem	In general, is there a problem with waiting in line for examination and/or analysis at the institutions where you receive health services?
Hospital Application	Where do you usually go first when you get sick?

## Empirical strategy

In the study, Microsoft Excel was used to organize the dataset, and Stata 16 was used for statistical analysis. Frequencies and percentages of individuals participating in the survey were calculated based on their satisfaction with examination services. The analysis process was carried out with a two-stage method. In the first stage, univariate analysis was applied to determine whether the independent variables were related to the dependent variable. In this context, Pearson’s chi-square independence test was used to evaluate whether the independent variables had a statistically significant relationship with satisfaction with health examination services. In the second stage, all independent variables were integrated into the same model and, considering the binary categorical structure of the dependent variable, a logit regression model (logit model) was applied to estimate the relationship between satisfaction with health examination services and the independent variables.

## Results

### Descriptive statistics and crosstabs

Percentage distributions and Pearson’s chi-square independence test results regarding the satisfaction of individuals with health examination services are presented in Table 3.

**Table 3:** Findings on factors affecting individuals’ satisfaction with medical examination services (N: 9502).

**Variables**	**Satisfaction with Health Examination Services (%)**			**n(%)**	***Prob*.**
		**No**	**Yes**		
**Sociodemographic Indicators**					
*Gender*					
	Male	48.9	46.6	47.5	0.032
	Female	51.1	53.4	52.5	
*Marital status*					
	Single	27.8	29.0	28.6	0.209
	Married	72.2	71.0	71.4	
*Educational Status*					
	No Degree	9.6	10.4	10.1	0.088
	Primary School	44.5	44.0	44.2	
	High School	23.1	23.0	23.0	
	University	20.9	20.0	20.3	
	Postgraduate	2.0	2.7	2.4	
*Income Satisfaction*					
	Satisfied	37.9	37.5	37.7	0.007
	Moderate	22.3	25.0	24.0	
	Not Satisfied	39.8	37.5	38.3	
**Individual Indicators**					
*Employment Status*					
	Employed	44.9	43.6	44.1	0.237
	Unemployed	55.1	56.4	55.9	
*SSI Registration*					
	Yes	85.7	87.8	87.0	0.003
	No	14.3	12.2	13.0	
*Happiness Level*					
	Very Happy	3.1	5.8	4.7	0.000
	Happy	48.9	48.0	48.3	
	Middle	32.8	34.0	33.5	
	Not Happy	11.9	9.9	10.7	
	Not Happy at All	3.3	2.3	2.7	
*Future Expectation*					
	It Will Be Better	21.8	23.9	23.1	0.002
	Will be the same	44.4	43.2	43.7	
	It Will Get Worse	27.9	25.7	26.5	
	No Idea	5.8	7.2	6.7	
*Health Status*					
	Yes	11.7	13.2	12.6	0.038
	No	88.3	86.8	87.4	
*Electronic Public Services Usage Status*					
	Yes	67.5	69.7	68.9	0.041
	No	25.5	24.3	24.7	
*General Health Services Satisfaction*					
	Satisfied	59.7	68.8	65.3	0.000
	Moderate	16.0	16.0	16.0	
	Not Satisfied	24.1	14.8	18.3	
	No Idea	0.2	0.4	0.3	
*Social Security Services Satisfaction*					
	Satisfied	60.6	62.1	61.5	0.000
	Moderate	14.8	12.2	13.2	
	Not Satisfied	10.7	7.2	8.5	
	No Idea	13.9	18.5	16.8	
**Institutional Indicators**					
*Appointment Problem*					
	Yes	47.9	48.2	48.0	0.019
	No	51.8	51.0	51.3	
	No Idea	0.4	0.9	0.7	
*Hygiene Problem*					
	Yes	25.7	22.1	23.5	0.000
	No	73.8	77.4	76.0	
	No Idea	0.5	0.6	0.5	
*Problem-Related to Doctor*					
	Yes	24.8	19.8	21.7	0.000
	No	74.6	79.7	77.8	
	No Idea	0.6	0.5	0.5	
*Adequacy of Number of Health Personnel*					
	Yes	19.4	40.7	32.6	0.000
	No	78.5	53.5	63.0	
	No Idea	2.1	5.8	4.4	
*High Test and Examination Fees*					
	Yes	42.1	53.0	48.8	0.000
	No	55.3	42.2	47.2	
	No Idea	2.6	4.8	4.0	
*The Problem of Drug Prices*					
	Yes	52.2	63.5	59.2	0.000
	No	46.6	33.8	38.7	
	No Idea	1.2	2.7	2.1	
*Queue Waiting Problem*					
	Yes	43.0	43.2	43.1	0.206
	No	56.4	55.8	56.0	
	No Idea	0.6	1.0	0.8	
*Hospital Application*					
	Primary Stage Health Organizations	35.1	39.6	37.9	0.000
	Second Step Health Organizations	61.7	55.6	57.9	
	Third Step Health Organizations	3.2	4.9	4.2	

According to the results of the chi-square test of independence, significant relationships were found between the satisfaction of individuals with examination services and sociodemographic-individual indicators and institutional indicators.

### Logit model results

The logit regression model was used in the study to analyze the effects of sociodemographic, individual and institutional factors on the satisfaction with examination services in health. The established model was found to be statistically significant (*P < 0.0001*). The Goodness of Fit Test results of the model are presented in Table 4.

**Table 4:** The goodness of fit test of the logit model.

Criteria	Logit
Pseudo R^2^	0.102
Cragg & Uhler’s R^2^	0.173
AIC	1.208
BIC	-75064.554
Area under ROC curve	0.716
Log likelihood	-5671.941
P-value	0.000
N	9502

According to Table 3, the Pseudo R^2^ value for the logit model is 0.102; The area under the ROC curve is 0.716 and the model is statistically significant. The estimation results of the logit regression model and the variance inflation factors (Vif) for the independent variables are presented in Table 5. To assess multicollinearity among the independent variables in the model, VIF values were examined, all of which were found to be below 5. This indicates that no multicollinearity problems exist among the independent variables [31].

**Table 5:** Logit regression model and variance inflation factors.

Variables	Coef.	Std. Err.	Prob.	Vif
**Sociodemographic Indicators**				
*Gender*				
Male (reference category)				
Female	0.021	0.052	0.695	1.34
*Age*	0.01	0.002	0	1.68
*Marital status*				
Single (reference category)				
Married	-0.152	0.054	0.005	1.13
*Educational Status*				
No degree	0.041	0.088	0.637	1.33
Primary School (reference category)				
High School	0.102	0.063	0.106	1.39
University	0.033	0.067	0.616	1.43
Postgraduate	0.478	0.158	0.003	1.09
*Income Satisfaction*				
Satisfied	0.02	0.059	0.732	1.61
Moderate	0.15	0.062	0.017	1.38
Not Satisfied (reference category)				
**Individual Indicators**				
*Employment Status*				
Employed	0.053	0.056	0.346	1.52
Unemployed (reference category)				
*SSI Registration*				
Yes	0.162	0.073	0.026	1.18
No (reference category)				
*Happiness Level*				
Very Happy	0.679	0.14	0	1.52
Happy	0.016	0.082	0.842	3.36
Middle	0.108	0.081	0.18	2.95
Not Happy (reference category)				
Not Happy at All	-0.082	0.15	0.587	1.23
*Future Expectation*				
It Will Be Better	0.134	0.07	0.054	1.68
Will be the same	0.055	0.058	0.342	1.66
It Will Get Worse (reference category)				
No Idea	0.238	0.104	0.022	1.26
*Health Status*				
Yes	0.135	0.072	0.061	1.1
No (reference category)				
*Interest in Health Issues*				
Interested	0.283	0.065	0	2.05
Moderate	0.115	0.074	0.119	1.96
Not Interested (reference category)				
No Idea	-0.287	0.212	0.176	1.12
*Electronic Public Services Usage Status*				
Yes	0.205	0.062	0.001	1.62
No (reference category)				
No Idea	-0.265	0.103	0.01	1.23
*General Health Services Satisfaction*				
Satisfied	0.607	0.069	0	2.19
Moderate	0.535	0.08	0	1.76
Not Satisfied (reference category)				
No Idea	1.257	0.471	0.008	1.03
*Social Security Services Satisfaction*				
Satisfied	0.195	0.09	0.029	3.95
Moderate	0.105	0.102	0.301	2.47
Not Satisfied (reference category)				
No Idea	0.429	0.101	0	2.88
**Institutional Indicators**				
*Appointment Problem*				
Yes	0.201	0.054	0	1.41
No (reference category)				
No Idea	0.594	0.345	0.085	1.07
*Hygiene Problem*				
Yes	-0.144	0.06	0.016	1.28
No (reference category)				
No Idea	-0.1	0.332	0.762	1.04
*Problem Related to Doctor*				
Yes	-0.315	0.059	0	1.18
No (reference category)				
No Idea	-0.549	0.318	0.085	1.03
*Adequacy of Number of Health Personnel*				
Yes	1.136	0.053	0	1.1
No				
No Idea	1.184	0.139	0	1.16
*High Test and Examination Fees*				
Yes	0.404	0.061	0	1.79
No (reference category)				
No Idea	0.466	0.151	0.002	1.48
*The Problem of Drug Prices*				
Yes	0.606	0.063	0	1.84
No (reference category)				
No Idea	0.843	0.217	0	1.52
*Queue Waiting Problem*				
Yes	-0.138	0.058	0.018	1.58
No (reference category)				
No Idea	-0.461	0.298	0.122	1.23
*Hospital Application*				
Primary Stage Health Organizations (reference category)				
Second Step Health Organizations	-0.139	0.048	0.004	1.1
Third Step Health Organizations	0.382	0.124	0.002	1.08
*Cons.*	-2.084	0.178	0	

Due to the nature of the discrete choice model family, quantitative interpretations of the coefficients will be made through marginal effects. Table 6 presents the marginal effects obtained from the model estimation.

**Table 6:** Marginal effect estimation results.

Variables	ey/dx	Std. Err.	Prob.	[95% Conf. Interval]	
**Sociodemographic Indicators**					
*Gender*					
Male (reference category)					
Female	0.008	0.020	0.695	-0.031	0.047
*Age*	0.004	0.001	0.000	0.002	0.005
*Marital status*					
Single (reference category)					
Married	-0.057	0.020	0.004	-0.096	-0.018
*Educational Status*					
No degree	0.016	0.034	0.635	-0.050	0.082
Primary School (reference category)					
High School	0.039	0.024	0.104	-0.008	0.086
University	0.013	0.026	0.615	-0.038	0.063
Postgraduate	0.164	0.048	0.001	0.070	0.258
*Income Satisfaction*					
Satisfied	0.008	0.023	0.732	-0.037	0.053
Moderate	0.056	0.023	0.016	0.011	0.102
Not Satisfied (reference category)					
**Individual Indicators**					
*Employment Status*					
Employed	0.020	0.021	0.345	-0.022	0.062
Unemployed (reference category)					
*SSI Registration*					
Yes	0.064	0.030	0.031	0.006	0.122
No (reference category)					
*Happiness Level*					
Very Happy	0.223	0.043	0.000	0.139	0.307
Happy	0.006	0.032	0.842	-0.057	0.070
Middle	0.042	0.032	0.187	-0.020	0.104
Not Happy (reference category)					
Not Happy at All	-0.033	0.062	0.592	-0.154	0.088
*Future Expectation*					
It Will Be Better	0.051	0.027	0.054	-0.001	0.103
Will be the same	0.022	0.023	0.344	-0.023	0.066
It Will Get Worse (reference category)					
No Idea	0.088	0.037	0.018	0.015	0.161
*Health Status*					
Yes	0.050	0.026	0.054	-0.001	0.101
No (reference category)					
*Interest in Health Issues*					
Interested	0.111	0.027	0.000	0.058	0.163
Moderate	0.047	0.030	0.121	-0.012	0.107
Not Interested (reference category)					
No Idea	-0.130	0.102	0.201	-0.329	0.069
*Electronic Public Services Usage Status*					
Yes	0.079	0.025	0.001	0.031	0.128
No (reference category)					
No Idea	-0.115	0.047	0.014	-0.207	-0.023
*General Health Services Satisfaction*					
Satisfied	0.254	0.031	0.000	0.193	0.315
Moderate	0.228	0.034	0.000	0.161	0.295
Not Satisfied (reference category)					
No Idea	0.444	0.113	0.000	0.223	0.666
*Social Security Services Satisfaction*					
Satisfied	0.079	0.038	0.036	0.005	0.152
Moderate	0.043	0.042	0.305	-0.040	0.126
Not Satisfied (reference category)					
No Idea	0.163	0.040	0.000	0.084	0.241
**Institutional Indicators**					
*Appointment Problem*					
Yes	0.077	0.021	0.000	0.036	0.117
No (reference category)					
No Idea	0.204	0.099	0.040	0.009	0.398
*Hygiene Problem*					
Yes	-0.056	0.024	0.018	-0.103	-0.010
No (reference category)					
No Idea	-0.039	0.131	0.768	-0.296	0.218
*Problem Related to Doctor*					
Yes	-0.126	0.025	0.000	-0.174	-0.078
No (reference category)					
No Idea	-0.232	0.153	0.129	-0.533	0.068
*Adequacy of Number of Health Personnel*					
Yes	0.391	0.017	0.000	0.358	0.424
No					
No Idea	0.402	0.033	0.000	0.336	0.467
*High Test and Examination Fees*					
Yes	0.155	0.023	0.000	0.109	0.200
No (reference category)					
No Idea	0.175	0.051	0.001	0.076	0.275
*The Problem of Drug Prices*					
Yes	0.240	0.026	0.000	0.189	0.290
No (reference category)					
No Idea	0.313	0.065	0.000	0.187	0.440
*Queue Waiting Problem*					
Yes	-0.053	0.022	0.018	-0.097	-0.009
No (reference category)					
No Idea	-0.192	0.138	0.166	-0.463	0.079
*Hospital Application*					
Primary Stage Health Organizations (reference category)					
Second Step Health Organizations	-0.053	0.018	0.004	-0.089	-0.017
Third Step Health Organizations	0.126	0.037	0.001	0.053	0.199

In terms of sociodemographic indicators, the study found that the probability of being satisfied with health examination services increases with age. Married individuals, however, are 5.7% less likely to be satisfied compared to single individuals. Education level also plays a significant role, with individuals holding postgraduate degrees being 16.4% more likely to express satisfaction compared to those with only primary education. Individuals registered with the Social Security Institution (SSI) are 6.4% more likely to be satisfied with health examination services than those without SSI registration. Satisfaction with income level also affects perceptions, as individuals moderately satisfied with their income are 5.6% more likely to be satisfied with medical examination services compared to those dissatisfied with their income.

In terms of individual indicators, individuals who are very happy with their lives overall are 22.3% more likely to be satisfied compared to those who are not happy. Individuals who expect the future to be better are 5.1% more likely to be satisfied with health examination services compared to those who expect the future to be worse. Individuals with a good general health status are 5% more likely to be satisfied with medical examination services than those with a poor health status. Similarly, individuals interested in health-related issues are 11.1% more likely to be satisfied compared to those who are not interested. The use of electronic public services also enhances satisfaction, with users being 7.9% more likely to be satisfied compared to non-users. Institutional indicators show strong associations with satisfaction. Individuals satisfied with general health services are 25.4% more likely to be satisfied with health examination services than those dissatisfied. Similarly, those satisfied with SSI services are 7.9% more likely to be satisfied. Individuals who experience appointment problems are 7.7% more likely to be satisfied with health examination services compared to those who do not. However, individuals who encounter hygiene problems are 5.6% less likely to be satisfied compared to those who do not experience such issues.

The findings obtained in the study support the generalideas. However, it is seen that the findings regarding the appointment problem are contrary to expectations. In this context, it is predicted that the finding that individuals with appointment problems are 7.7% more likely to be satisfied with health examination services compared to those without appointment problems may be related to the developments in Türkiye’s health policy and system. In the historical background of the Turkish health system, the referral system is among the practices planned to be implemented in line with the 2003 Health Transformation Program. In this regard, the referral system was piloted in certain provinces (Isparta, Denizli) in 2005, but was later abolished for various reasons. This situation caused overcrowding in health institutions during the relevant period and in the following period, and thus caused appointment problems. These problems led to various wastes in time and cost savings, which is one of the main objectives of the health system, and created obstacles in the access of essential patients to health services. In this context, various initiatives have been taken to solve appointment problems (approved appointment period, ensuring service integration between institutions by opening a quota for family physicians in the central appointment system, developing an application (healthy solution) to solve appointment problems). As a result of these initiatives, it is envisaged that people facing appointment problems will have easier access to services. It is thought that these facilitations may increase the satisfaction of individuals. Contrary to the existing views, the finding obtained in the study supports the relatedidea.

In terms of health facility indicators, the study reveals several significant factors affecting satisfaction with health examination services. Individuals who report having a problem with their doctor are 12.6% less likely to be satisfied compared to those who do not. Individuals who find the number of health personnel sufficient are 39.1% more likely to be satisfied with health examination services compared to those who do not. Individuals who consider analysis and examination fees to be high are 15.5% more likely to be satisfied compared to those who do not find the fees high. Similarly, individuals who perceive problems with medicine prices are 24% more likely to be satisfied with health examination services compared to those who do not report such concerns. Waiting times also impact satisfaction; individuals who experience problems with waiting in queues are 5.3% less likely to be satisfied compared to those who do not face such issues. Individuals who apply to secondary healthcare institutions are 5.3% less likely to be satisfied with health examination services compared to those who apply to primary healthcare institutions, while individuals who apply to tertiary healthcare institutions are 12.6% more likely to be satisfied with health examination services compared to those who apply to primary healthcare institutions.

The findings of the study generally support the expectations. However, the results on the prices of analysis and medicines are contrary to expectations. The possible reasons for this situation are analyzed in detail and presented below.

The rate of satisfaction with health services is 15.5% higher among those who find the fees for tests and examinations high. It is thought that this finding may be related to Türkiye’s health financing system. In this context, health services in public health institutions are financed by the General Health Insurance, which started to be implemented in 2012. General Health Insurance finances a certain proportion of the services received from private health institutions as well as public institutions. In this regard, it is predicted that the findings of this study may be related to the people who receive services from private health institutions, and in addition, financing their services through the insurance they have may increase their satisfaction.

The finding on drug purchases may be related to Türkiye’s health financing practices. While a certain percentage of drug purchase payments are financed by insurance, a certain percentage and examination fees are collected from pharmacies through out-of-pocket payments. However, the financing of health services for people who are unable to pay and who experience difficulties in this sense is covered by the state. These people are determined on the basis of their monthly income being below one-third of the minimum wage, and are facilitated in the financing of service and medicine purchases. Therefore, it is predicted that individuals with high service satisfaction despite their low drug purchasing power may benefit from the facilities in health financing policies and their satisfaction may stem from this situation.

## Discussion

Satisfaction in health is a topic that has been extensively researched, with ongoing efforts to achieve continuous improvement. In this regard, globalization and advancements in communication technologies have facilitated the emergence and evolution of the concept of health satisfaction, which can now be assessed through various factors, including the quality, duration, and cost of services received. Considering the historical process of health services, being able to receive treatment and access to services were considered as a factor of satisfaction, while today this situation has changed. In addition to the factors mentioned, the recent global pandemic (covid-19) is also of great importance. Therefore, by examining satisfaction with health examination services in the post-pandemic period, this study aimed to provide significant recommendations for health policy and system designers, contributing to positive outcomes in healthcare.

The analysis of representative data for Türkiye revealed that factors such as age, marital status, education level, social security enrollment, income satisfaction, happiness level, future expectations, health status, interest in health issues, use of electronic public services, satisfaction with general health services, satisfaction with social security services, appointment problems, hygiene issues, doctor-related problems, adequacy of health personnel, high analysis and examination fees, drug price concerns, waiting in line, and type of hospital application significantly influence satisfaction with health examination services. Furthermore, the study found that 61.85% of Turkish citizens report being satisfied with health examination services.

The probability of being satisfied with health examination services was found to increase with age. This result is thought to stem from the ability of older individuals to compare past and present healthcare services, leading to a greater accumulation of knowledge over time. On the other hand, it is suggested that Türkiye’s ‘Home Health Services’ system may contribute to this outcome. As per Directive No. 3895, issued on February 1, 2010, home health services were expanded to all 81 provinces in Türkiye [32]. These services are designed not only for the elderly but also for patients unable to visit healthcare facilities, including those with chronic conditions, disabilities, or recovering from illness. In Türkiye, home health services originated in 1930 with the enactment of the Law No. 1593 on Public Hygiene [33]. These services were later integrated into the 1963 Development Plan, which called for the establishment of healthcare systems prioritizing home and outpatient care as cost-effective alternatives to hospital treatments that benefit a limited segment of the population [34]. The first home care project was initiated in Istanbul, Ankara, Adana, and Izmir in 1993, and by 2004, all public hospitals began offering home health services [35]. This situation shows that the service, which continues to be implemented today, has continued to improve from the historical past to the present. As a matter of fact, the finding obtained supports this situation. The finding that satisfaction increases with increasing age is equivalent to the results of a study in the literature conducted in 2009 [4].

Married individuals are less likely to be satisfied with health examination services compared to single individuals. This disparity may be linked to differing health demands between these groups. Married individuals often seek healthcare not only for themselves but also for their spouses and, as parents, for their children. This broader responsibility leads to greater scrutiny and higher expectations of health services. For example, parents may exhibit increased attentiveness and selectivity in the care provided during their children’s illness, which can influence their overall satisfaction with health services. Based on the results obtained in the study, it is seen that the mentioned role may have an impact on satisfaction.

A higher level of education was found to increase the likelihood of satisfaction with health examination services. This finding may be related to the correlation between higher education and improved health literacy. It is a known fact that as individuals attain higher levels of education, they are better equipped to navigate health demand processes effectively. Although Türkiye provides health services consistently across all regions and provinces, variations in health outcomes and mortality rates highlight the importance of health literacy in optimizing these services. Managing health demands and therefore health preferences with the right health institution, outpatient clinic and physician saves time in terms of diseases and paves the way for positive results in health problems. Therefore, it is an expected result that higher education levels correlate with greater satisfaction in healthcare. The findings of this study support this expectation and are consistent with the results of the studies in the literature [4,10].

SSI enrollment and satisfaction with SSI services were found to increase the likelihood of satisfaction with health examination services. Historically, the Turkish health system has made significant efforts to establish social security and ensure access to healthcare for all segments of the population. Although not all services are provided entirely free of charge (as in a socialist health system), individuals are assured access to healthcare with minimal co-payments. The social security of those who cannot afford to pay the co-payment (people whose monthly income is less than 1/3 of the minimum wage) is financed by the ‘Green Card’ in the historical past and the ‘General Health Insurance’ today. However, rising costs in healthcare services, such as private healthcare fees and drug prices, may impose limitations on access for some individuals. Despite these challenges, social security has played a crucial role in facilitating easier access to healthcare services. The findings of this study highlight the importance of social security in healthcare and are consistent with results of studies in the literature [24,14].

Satisfaction with income level was found to increase the likelihood of being satisfied with health examination services. Income serves as a critical determinant of individuals’ demand for healthcare services. In this context, individuals have the option to access services not only from public hospitals but also from private hospitals and physicians in various provinces. Similarly, health services can be requested from a different country where there is a service suitable for the health problem. However, such services and expenses incurred through health tourism are not covered by social security, meaning that access to these options is shaped by individuals’ income levels. Therefore, higher income levels are expected to enhance satisfaction with healthcare services. The findings of this study align with this expectation.

Being a happy individual and having a positive expectation for the future were found to increase the likelihood of satisfaction with health examination services. A positive mood and outlook on life can affect health outcomes positively. Individuals who maintain optimism and actively seek solutions to health problems are better equipped to manage their health processes effectively. This proactive approach can lead to a smoother and faster recovery process. It is expected that individuals who continue their lives in this way will be more satisfied with health services. The findings obtained in the research are consistent with the said expectation.

Good health status also increased the likelihood of satisfaction with health examination services. Individuals in good health may approach healthcare interactions with greater ease and cooperation, as they do not face severe health challenges. This fosters effective communication between doctors and patients, resulting in positive health outcomes. Such dynamics streamline service acquisition processes and promote faster recovery, leading to greater satisfaction with health services. The findings of this study, along with previous research, support this conclusion [13].

Interest in health issues was found to increase the likelihood of satisfaction with health examination services. This finding may be related to individuals’ health literacy levels, as higher literacy enables them to manage health-related processes more effectively. The critical role of literacy in health services has recently gained attention, prompting the Ministry to launch a study on health literacy in 2023 [36]. Based on the results to be obtained in the relevant study, it is predicted that the health service process can be managed correctly, positive health outcomes can be achieved, and satisfaction can be increased. The findings of this study are consistent with existing literature, which indicates that limited health literacy is associated with lower satisfaction [12].

The use of electronic public services was found to increase the likelihood of satisfaction with health examination services. This finding may be associated with the role of digital hospitals in Türkiye’s healthcare system. Within the framework of the Electronic Health Record Adoption Model, hospitals in Türkiye are assessed across 8 stages of digitalization, with those achieving levels 6 and 7 awarded accreditation certificates [37]. This transition to digital processes has significantly reduced paper use and accelerated the delivery of services through digital platforms. It is thought that this situation may influence increasing satisfaction in health services. The findings reached in the study support this idea.

Experiencing cleaning or hygiene problems was found to decrease the likelihood of satisfaction with health examination services. This finding may be affected by the timing of the study, which was conducted in the post-pandemic period. The pandemic underscored the critical importance of hygiene, shaping individuals’ health perceptions and increasing their sensitivity to cleanliness in healthcare settings. Additionally, the role of hygiene issues as a primary driver of epidemics may have further heightened awareness and expectations regarding cleanliness. Therefore, it is predicted that the attention paid to cleaning due to reasons such as health problems and fear experienced during the pandemic process may be important in terms of people’s satisfaction. In this regard, the result reached in the study supports the mentioned prediction.

The presence of problems related to doctor behavior was found to decrease the likelihood of satisfaction with health examination services. This finding may be related to communication problems during the healthcare process. Such problems are often a contributing factor to violence in healthcare settings and can lead to behavioral changes on both sides during the service interaction. It is predicted that these communication problems significantly affect satisfaction with health services. The findings obtained support this prediction.

Perceiving the number of doctors and health personnel as sufficient was found to increase the likelihood of satisfaction with health examination services. Preferences regarding doctors and health personnel are crucial factors affecting satisfaction with healthcare. Advancements in technology, increasing health literacy levels, and platforms for physician selection and evaluation (e.g., RateMDs) play a significant role in this context. These tools enable individuals to make informed choices about healthcare institutions and personnel. In this direction, it is predicted that individuals who actively select their physicians are more likely to be satisfied with health services. The findings of this study align with this prediction.

Experiencing problems related to waiting in line for an examination or test was found to decrease the likelihood of satisfaction with health examination services. The lack of a mandatory primary healthcare referral system in Türkiye’s health system has contributed to certain issues, such as overcrowding in secondary and tertiary health institutions and appointment backlogs. The ability of individuals to directly access tertiary health services without primary care referrals, coupled with their sociodemographic characteristics, can limit appointment availability for critical patients, increase waiting times, delay disease management, and lead to negative health outcomes. These factors are expected to reduce satisfaction with health services, and the study findings confirm this expectation. To address these issues, initiatives have been introduced, including a directive from the Social Security Institution in 2022 requiring private hospitals to make all their services accessible to patients with social security coverage [38]. In addition, the ‘Approved Appointment System’, launched in 2024, allows patients to confirm or cancel their appointments [3]. Related initiatives also reveal the role of waiting times on satisfaction. The findings of this study align with these developments and are consistent with results reported in the literature [10,14,16].

Regarding hospital applications, individuals who apply to secondary healthcare institutions are less likely to be satisfied with health examination services compared to those who apply to primary healthcare institutions. On the other hand, individuals who apply to tertiary healthcare institutions are more likely to be satisfied compared to those who use primary healthcare institutions. These findings may be associated with systemic changes in Türkiye’s healthcare structure. The opening of the first city hospital (Yozgat City Hospital) in 2016 and the subsequent closure of many state hospitals to ensure the occupancy rates of city hospitals play a significant role in this context. The reduction in the availability of secondary healthcare institutions may have led to negative perceptions of such facilities. At the same time, the redirection of individuals to tertiary hospitals, including city hospitals, may have fostered a perception of superior healthcare services being provided at these institutions. These dynamics, as reflected in the study results, suggest that differences in satisfaction levels may stem from these systemic changes. The findings are consistent with results reported in the literature [16].

The pandemic and the severe health problems that followed have significantly influenced individuals’ perceptions of healthcare, creating a patient profile that critically evaluates each stage of the service process. The fear of re-experiencing similar health crises has also driven patients to be more selective in their healthcare demands. In addition, it is thought that problems and intensity experienced in appointment problems, and situations such as being able to find an appointment for a later date are also determinants of satisfaction. It is seen that the increase in the number of beds and healthcare personnel following the opening of city hospitals in Türkiye in line with the ‘Health Transformation Program’ also plays an important role in healthcare examination satisfaction within the scope of institutional variables. However, the closure of second level (state) hospitals in certain cities to ensure the capacity utilization of city hospitals, along with the redirection of patients to primary and tertiary healthcare institutions, also holds significant importance in shaping examination satisfaction, which is the focus of this study. In this regard, it is observed that the necessary importance is given to the health institutions/services at the relevant level, that primary health institutions provide services as A, B, C, D groups, and that A and B group family health centers provide services with flexible working hours [39] are also important in health examination services, and this importance is also supported by the study results.

## Conclusion

The most important factor for health services to be carried out in a desired and correct manner is healthy communication. In this context, the concept of satisfaction plays a pivotal role in fostering healthy communication between physicians and patients or their relatives. Patients who are satisfied with the services, institutions, and personnel are more likely to articulate their health needs clearly and approach the service process with greater moderation. In fact, the opposite can also occur, and negative results can be encountered in health services. Therefore, it is thought that health strategies, policies and therefore the system play an important role. This study, which focuses on satisfaction with health examination services in Türkiye, aims to interpret the findings considering current practices and policies within the healthcare system regarding examination services.

In the study, the micro dataset of the 2023 Life Satisfaction Survey was used. Health examination services were analyzed in relation to various variables. However, it is thought that a more transparent perspective on the system can be provided by evaluating the results of certain variables under a common heading to draw a general framework. Accordingly, the variables included in the study were categorized under three main headings: sociodemographic, individual, and institutional.

Within the scope of sociodemographic factors, satisfaction with health examination services was analyzed based on gender, age, marital status, education, and income. The findings indicate that single individuals are more likely to be satisfied with health examination services as their age, education, and income increase. This result is thought to be associated with structural differences in Türkiye’s healthcare system. In this regard, it is predicted that improvements in services from the past to the present, initiatives for quality health services and the practices put into effect within the scope of the ‘Health Transformation Program’ are decisive. These practices are thought to enhance satisfaction by facilitating positive outcomes in individuals’ healthcare demands.

Within the scope of individual factors, the study examined health examination satisfaction in relation to employment status, SSI registration and satisfaction with SSI services, happiness level, future expectations, health status, interest in health issues, use of electronic public services, and satisfaction with general health services. The findings reveal that having good health, being a happy individual, showing interest in health issues, being registered with SSI, satisfaction with SSI services, and using electronic public services increase the likelihood of satisfaction with health examination services. It is predicted that the obtained result may be related to many factors. Specifically, the absence of significant health problems, a positive outlook on life, effective communication during health issues, financial ease of access to health services through the General Health Insurance (GSS), and the ability to utilize digital health service applications (e.g., e-Pulse, MHRS) are likely to play important roles. Thus, the findings are considered closely related to these factors.

Within the scope of institutional factors, health examination satisfaction was evaluated based on appointment issues, hygiene problems, doctor-related issues, the adequacy of health personnel, high test and examination fees, drug price problems, waiting times, and hospital applications. According to the research findings, the results obtained under institutional variables vary significantly. Specifically, experiencing cleaning and hygiene issues, insufficient numbers of doctors and health personnel, and delays caused by waiting in line for examinations or tests decrease the likelihood of being satisfied with health examination services. On the other hand, perceiving the number of doctors and health personnel as sufficient increases satisfaction. Furthermore, when satisfaction is analyzed based on hospital applications, individuals utilizing secondary healthcare institutions are less likely to be satisfied compared to those using primary healthcare services, whereas those accessing tertiary healthcare institutions are more likely to be satisfied compared to primary care users. It is thought that the results regarding the decrease in satisfaction in the study may be related to the post-pandemic period in which the study was conducted.

## Policy recommendations

Based on the findings of this study, several suggestions can be made for future research. The results regarding the step system in health services highlight the critical role of preventive health services within the healthcare system. Managing the health service process varies depending on the socio-demographic characteristics of individuals, which can lead to reduced satisfaction with health examination services, as well as negative health outcomes and an increased financial burden on the system. In this context, health policies and practices can be designed to better guide individuals within the healthcare system. In this regard, it is thought that health policies can be designed, and practices can be developed to guide people in the health system. On the other hand, in this study conducted in the post-pandemic period, it is also emphasized that there is a trust problem in the health system. It is anticipated that a lack of trust negatively impacts the service process and decreases satisfaction. Future studies and initiatives could focus on rebuilding and strengthening trust in the health system. Furthermore, the findings underline the importance of digitalization and digital hospitals in health services. Satisfaction with health examination services can be further enhanced by advancing digital health initiatives and expanding digital service offerings.

The present study utilizes a secondary dataset derived from the 2023 Life Satisfaction Survey. While the survey provides a range of questions that are applicable and relevant for the purposes of the analysis, its design may not encompass all pertinent variables influencing Satisfaction with Health Examination Services. For instance, it does not explicitly account for distinctions between urban and rural populations. Furthermore, the nature of the dataset and the methodology employed do not allow for the establishment of causal relationships.

## References

[1] Beyatlı H. Hastane ve sağlık işletmeleri yönetimi. Ankara: Nobel Akademik Yayıncılık. 2017.

[2] Özel B. Erişim tarihi: 28.10.2024. 2024. Available from: https://www.hurriyet.com.tr/ekonomi/sgkdan-ozel-hastane-karari-42074664

[3] Türkay Ş. Erişim tarihi: 28.10.2024. 2024. Available from: https://www.aa.com.tr/tr/saglik/hastanelerde-onayli-randevu-donemi-basladi/3217739

[4] Ho C-K. Satisfaction with Taiwan’s medical institutions of research and analysis. Journal of Statistics and Management Systems. 2009;12(5):949–61. doi: 10.1080/09720510.2009.10701433

[5] Maseko FC, Chirwa ML, Muula AS. Client satisfaction with cervical cancer screening in Malawi. BMC Health Serv Res. 2014;14:420. doi: 10.1186/1472-6963-14-420 25245860 PMC4180310

[6] Selmouni F, Zidouh A, Alvarez-Plaza C, El Rhazi K. Perception and satisfaction of cervical cancer screening by Visual Inspection with Acetic acid (VIA) at Meknes-Tafilalet Region, Morocco: a population-based cross-sectional study. BMC Womens Health. 2015;15:106. doi: 10.1186/s12905-015-0268-0 26597844 PMC4657367

[7] Hitzig SL, Dilkas S, Payne MW, MacKay C, Viana R, Devlin M, et al. Examination of social disconnectedness and perceived social isolation on health and life satisfaction in community-dwelling adults with dysvascular lower limb loss. Prosthet Orthot Int. 2022;46(2):155–63. doi: 10.1097/PXR.0000000000000069 35412523

[8] Alhaqbani SM, Bawazir AA. Assessment of Pregnant Women’s Satisfaction with Model of Care Initiative: Antenatal Care Service at Primary Health Care in Cluster One in Riyadh, Saudi Arabia. Healthcare (Basel). 2022;10(1):151. doi: 10.3390/healthcare10010151 35052314 PMC8775455

[9] Charters E, Khom MJ, Baker J, Lindsay T. Patient satisfaction and cost analysis of telehealth delivered by allied health oncology clinicians. Contemp Oncol (Pozn). 2022;26(1):44–8. doi: 10.5114/wo.2022.115047 35506032 PMC9052344

[10] Efe YS, Erdem E, Doğan M, Bağcı K, Öztürk S, Öztürk MA. Anxiety and healthcare satisfaction of mothers with children hospitalized in the pediatric emergency service. Arch Pediatr. 2022;29(3):207–12. doi: 10.1016/j.arcped.2022.01.007 35094906

[11] Ginthotavidana SSC, Waidyasekara KGAS. A performance measurement model for the housekeeping services in healthcare facilities. Facilities. 2022;40(1/2):56–75.

[12] Mefford MT, Zhou H, Fan D, Fang MC, Prasad PA, Go AS, et al. Health Literacy and Treatment Satisfaction Among Patients with Venous Thromboembolism. J Gen Intern Med. 2023;38(7):1585–92. doi: 10.1007/s11606-022-07852-3 36326991 PMC10212857

[13] Parinyarux P, Yotsombut K. Customers’ satisfaction toward drugstore facilities and services based on the good pharmacy practice standard in Thailand. Pharm Pract (Granada). 2022;20(1):2601. doi: 10.18549/PharmPract.2022.1.2601 35497907 PMC9014892

[14] Sakti D, Firdaus A, Utami T, Jati K, Mahayana I, Wardhana F, et al. Patients’ satisfaction with ophthalmology clinic services in a public teaching hospital. Patient Prefer Adherence. 2022;723–35. 35340758 10.2147/PPA.S347394 PMC8943654

[15] Zikusooka M, Hanna R, Malaj A, Ertem M, Elci OC. Factors affecting patient satisfaction in refugee health centers in Turkey. PLoS One. 2022;17(9):e0274316. doi: 10.1371/journal.pone.0274316 36112570 PMC9480993

[16] Heri R, Yahya-Malima KI, Malqvist M, Mselle LT. Women’s expectations of and satisfaction with antenatal care services in a semi-urban setting in Tanzania and associated factors: A cross-sectional survey. Healthcare. 2023;11(16):2321. 37628519 10.3390/healthcare11162321 PMC10454190

[17] World Health Assembly. Fifty-fifthe World Health Assembly, Geneva, 13-18 May 2002: resolutions and decisions, annexes. World Health Organization; 2002 [cited April 2025]. Available from: http://www.who.int/iris/handle/10665/259364

[18] Regulation on Non-Sanitary Establishments. Official Gazette. No: 22416. 1995.

[19] Spatial Plans Construction Regulation. Official Gazette. Issue: 29030. 2014.

[20] Regulation on Environmental Impact Assessment. Official Gazette. Issue: 31907. 2022.

[21] Gajewska P, Piskrzyńska K. Measuring quality of maternity services using the servqual method. Reg Form Dev Stud. 2016;3:50–9. doi: 10.15181/rfds.v20i3.1343

[22] Andersen R. A Behavioral Model of Families’ Use of Health Services. Research Series #25, The University of Chicago, Center for Health Administration Studies, Chicago. 1968.

[23] Magadi JP, Magadi MA. Ethnic inequalities in patient satisfaction with primary health care in England: Evidence from recent General Practitioner Patient Surveys (GPPS). PLoS One. 2022;17(12):e0270775. doi: 10.1371/journal.pone.0270775 36542601 PMC9770381

[24] Mohammed S, Souares A, Lorenzo Bermejo J, Babale SM, Sauerborn R, Dong H. Satisfaction with the level and type of resource use of a health insurance scheme in Nigeria: health management organizations’ perspectives. Int J Health Plann Manage. 2014;29(4):e309-28. doi: 10.1002/hpm.2219 24301516

[25] Kodjebacheva GD, Culinski T, Kawser B, Coffer K. Satisfaction With Telehealth Services Compared With Nontelehealth Services Among Pediatric Patients and Their Caregivers: Systematic Review of the Literature. JMIR Pediatr Parent. 2023;6:e41554. doi: 10.2196/41554 37000504 PMC10176140

[26] Berbaum KS, Franken EA Jr, Dorfman DD, Caldwell RT, Krupinski EA. Role of faulty decision making in the satisfaction of search effect in chest radiography. Acad Radiol. 2000;7(12):1098–106. doi: 10.1016/s1076-6332(00)80063-x 11131054

[27] Krupinski EA, Berbaum KS, Schartz KM, Caldwell RT, Madsen MT. The Impact of Fatigue on Satisfaction of Search in Chest Radiography. Acad Radiol. 2017;24(9):1058–63. doi: 10.1016/j.acra.2017.03.021 28549868 PMC5557668

[28] Myszewski JM, Sinha M. A model for determining the value of patient satisfaction in healthcare. BPMJ. 2019;26(3):798–815. doi: 10.1108/bpmj-03-2019-0123

[29] Kim D, Cho J. Analysis of the health examination service process using service blueprint: Focus on the older adult patient in South Korea. Healthcare. 2023;11(20):2709. 37893782 10.3390/healthcare11202709 PMC10606208

[30] Turkstat. 2023 [cited November 03 2024]. Available from: http://www.tuik.gov.tr/MicroVeri/YMA_2023/english/index.html

[31] Çebi Karaaslan K. Analysis of Factors Affecting Individuals’ Sources of Happiness with Multinomial Logistic Model. Eğitimde ve Psikolojide Ölçme ve Değerlendirme Dergisi. 2021;12(3):286–302. doi: 10.21031/epod.925631

[32] Evde Sağlık Hizmetleri Yönergesi. Sağlık Bakanlığınca Sunulan Evde Sağlık Hizmetlerinin Uygulama Usul ve Esasları Hakkında Yönerge. Sayı: 3895. 2010.

[33] Umumi Hıfzıssıhha Kanunu. 6/5/1930 tarihli Resmi Gazete, Sayı: 1489, Tertip: 3 Cilt: 11 Sayfa: 143. 1930.

[34] Planı K. Kalkınma Planı 1963-1967. DPT: Başbakanlık. 1963.

[35] Altuntaş M, Yılmazer T, Güçlü Y, Öngel K. Evde sağlık hizmeti ve günümüzdeki uygulama şekilleri. Tepecik Eğit Hast Derg. 2010;20:153–8.

[36] Çalık Göçümlü B. 15 binden fazla hanede “sağlık okuryazarlığı” araştırması başlatıldı (aa.com.tr) Erişim Tarihi: 10.03.2024. 2023.

[37] Sağlık Bakanlığı TC. Erişim Tarihi: 30.10.2024. Available from: https://dijitalhastane.saglik.gov.tr/TR-4858/emram-hakkinda.html

[38] Özel B. Erişim tarihi: 28.10.2024. 2024. Available from: https://www.hurriyet.com.tr/ekonomi/sgkdan-ozel-hastane-karari-42074664

[39] Aile Hekimliği Uygulama Yönetmeliği. Erişim tarihi: 08.10.2024. T.C. Sağlık Bakanlığı. 2015. Available from: https://www.mevzuat.gov.tr/Metin.Aspx?MevzuatKod=7.5.17051&MevzuatIliski=0&sourceXmlSearch=aile%20he

