# Correction: Understanding public perception of health examination services: Key factors influencing satisfaction in Türkiye

**DOI:** 10.1371/journal.pone.0331501

**Published:** 2025-09-03

**Authors:** Kübranur Çebi Karaaslan, Hülya Diğer, Tubanur Çebi

There are errors in the author affiliations. The correct affiliations are as follows:

Kübranur Çebi Karaaslan^1^, Hülya Diğer^2^, Tubanur Çebi^3^

**1** Department of Econometrics, Faculty of Economics and Administrative Sciences, Economic and Social Research Application and Research Center, Erzurum Technical University, Türkiye, **2** Department of Health Management, Faculty of Economics and Administrative Sciences, Economic and Social Research Application and Research Center, Erzurum Technical University, Türkiye, **3** Department of Dental Prosthesis Technology, The Health Services Vocational School, Atatürk University, Erzurum, Türkiye.
